# Hierarchical core-shell NiCo_2_O_4_@NiMoO_4_ nanowires grown on carbon cloth as integrated electrode for high-performance supercapacitors

**DOI:** 10.1038/srep31465

**Published:** 2016-08-12

**Authors:** Liang Huang, Wei Zhang, Jinwei Xiang, Henghui Xu, Guolong Li, Yunhui Huang

**Affiliations:** 1 State Key Laboratory of Material Processing and Die & Mould Technology, School of Materials Science and Engineering, Huazhong University of Science and Technology, Wuhan 430074, China

## Abstract

Hierarchical core-shell NiCo_2_O_4_@NiMoO_4_ nanowires were grown on carbon cloth (CC@NiCo_2_O_4_@NiMoO_4_) by a two-step hydrothermal route to fabricate a flexible binder-free electrode. The prepared CC@NiCo_2_O_4_@NiMoO_4_ integrated electrode was directly used as an electrode for faradaic supercapacitor. It shows a high areal capacitance of 2.917 F cm^−2^ at 2 mA cm^−2^ and excellent cycling stability with 90.6% retention over 2000 cycles at a high current density of 20 mA cm^−2^. The superior specific capacitance, rate and cycling performance can be ascribed to the fast transferring path for electrons and ions, synergic effect and the stability of the hierarchical core-shell structure.

In recent years, renewable and clean energy storage technologies have attracted considerable attention due to the serious environmental pollution and fossil-fuel energy crisis^1^,^2^,^3^,^4^,^5^,^6^. Furthermore, the extensive demand of electric vehicles and portable electronic devices promote humans to develop more new energy storage devices^7^,^8^,^9^. Lithium-ion batteries^10^, sodium-ion batteries^11^,^12^ and supercapacitors (SCs)^13^ are potential energy storage devices currently. Among them, SCs have competitive characteristics such as high power density, long cycle life, fast charging/discharging rate, good safety and low maintenance cost^14^. Although the carbonaceous materials such as active carbon^15^, porous carbon^16^, graphene^17^,^18^, and carbon nanotubes^19^ can deliver a high power density, the electrical double layer supercapacitive (EDLS) mechanism gives rise to their low specific capacitance and energy density. So the researchers mainly focus on developing faradaic electrode materials that have higher capacity and larger energy density. In the past few years, various faradaic electrode materials, including transition metal oxides^20^, hydroxides^21^,^22^, sulfides^23^,^24^ and conductive polymers^25^,^26^ were intensively developed. For example, MnO_2_^27^,^28^, NiO^29^ and Co_3_O_4_^30^ are some of the most notable faradaic electrode materials, but their intrinsic electrical conductivities are poor. Conductive polymers such as polyaniline^26^, polypyrrole^31^,^32^ and Poly (3, 4-ethylenedioxythiophene)^33^ can offer a high specific capacity, but the volume expansion leads to the poor cycling performance.

Currently, binary transition metal oxides such as NiCo_2_O_4_^34^,^35^,^36^,^37^,^38^, NiMoO_4_^39^,^40^,^41^,^42^,^43^ and CoMoO_4_^44^,^45^ have been attracted much attention as electrode materials for supercapacitors because of their high electrical conductivity, remarkable specific capacity and environmental compatibility. For example, Lou *et al*.^46^ fabricated the NiCo_2_O_4_ nanorods and nanosheets on the carbon fiber through a facile solution method, and they delivered high capacitances of 1023.6 and 1002 F g^−1^, respectively. Huang *et al*.^42^ reported a Ni foam supported NiMoO_4_ nanoplate arrays integrated electrode, showing a high specific areal capacitance of 3.4 F cm^−2^ at 2 mA cm^−2^. However, in order to further improve the electrochemical performance, combining two types of binary metal oxides to form a unique hierarchical nanostructure is an efficient way. For example, Mai *et al*.^45^ reported hierarchical MnMoO_4_/CoMoO_4_ heterostructured nanowires, which showed an enhanced supercapacitive performance.

In addition, core-shell nanostructure electrode materials can effectively improve electrochemical performance^47^. For example, Cai *et al*.^48^ reported a manganese oxide/carbon york-shell anode for lithium-ion batteries, presenting a high capacity and excellent cycling performance at a high current density. Rational structure design of electrode is also important to its electrochemical performance. An efficient way is to synthesize binder-free designed electrode, because the host materials can directly connect with the conductive substrate. Carbon cloth is usually used as collector due to its low cost, high conductivity and flexibility^49^,^50^.

Herein, we fabricated hierarchical core-shell NiCo_2_O_4_@NiMoO_4_ nanowires grown on carbon cloth to form CC@NiCo_2_O_4_@NiMoO_4_ composite by a two-step hydrothermal route and evaluated as a flexible integrated electrode for supercapacitor. Apparently, this unique hybrid structure has some advantages: (1) the synergic effect of NiCo_2_O_4_ and NiMoO_4_ can further improve the electrochemical performance; (2) the binder-free architecture can provide a fast electron transfer path, efficiently enhancing the rate performance; (3) the core-shell nanostructure is more stable, giving an excellent cycling stability at high current density. The as-fabricated carbon cloth supported hierarchical core-shell NiCo_2_O_4_@NiMoO_4_ wires provide very high areal specific capacitance (ASC) of 2.917 F cm^−2^ at 2 mA cm^−2^. Moreover, the CC@NiCo_2_O_4_@NiMoO_4_ integrated electrode exhibits an excellent cyclabilty with 90.6% retention over 2000 cycles.

## Results and Discussion

**Figure 1:**
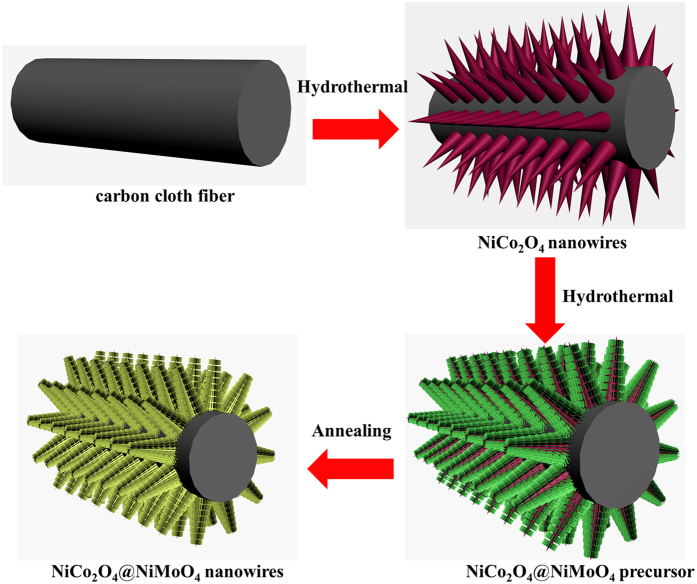
Schematic formation process of CC@NiCo_2_O_4_@NiMoO_4_ integrated electrode.

The schematic preparation process of CC@NiCo_2_O_4_@NiMoO_4_ is presented in Fig. 1. In this work, the fabrication of integrated CC@NiCo_2_O_4_@NiMoO_4_ electrode contains two steps. Firstly, the aligned pristine NiCo_2_O_4_ nanowires were grown on a piece of carbon cloth to fabricate the CC@NiCo_2_O_4_ backbone by a facile hydrothermal reaction and post-annealing process. Afterwards, the interconnected tiny NiMoO_4_ nanosheets were uniformly deposited on the CC@NiCo_2_O_4_ backbone by a secondary hydrothermal process. Finally, the precursor was transformed to hierarchical core-shell CC@NiCo_2_O_4_@NiMoO_4_ integrated electrode through an annealing process in the pure N_2_ atmosphere.

X-ray diffraction (XRD) was used to check the phase of as-prepared samples, as displayed in Fig. S1. We directly used the binder-free samples for XRD test. The patterns indicate that the pure carbon cloth has two obvious unique characteristic peak of carbon^49^. Additionally, except for the diffraction peak of carbon, all the identified peaks can be well indexed to cubic spinel NiCo_2_O_4_ (JCPDS no. 20-0781) and monoclinic NiMoO_4_ phase (JCPDS no. 86-0361).

**Figure 2:**
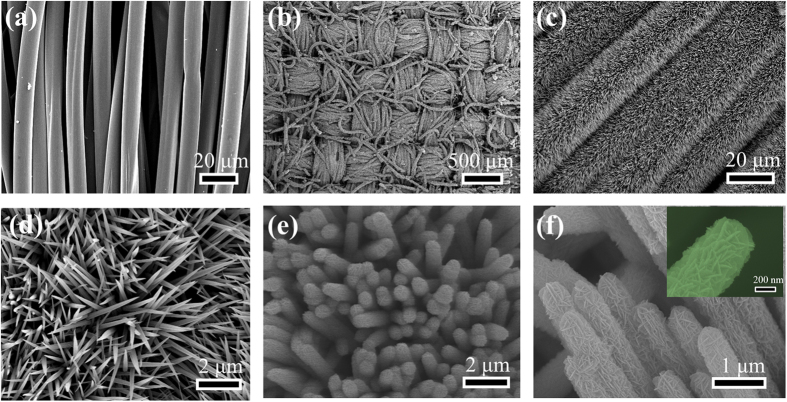
SEM analysis results of as prepared electrode materials. (**a**) SEM image of bare carbon cloth. (**b–d**) SEM images of NiCo_2_O_4_ nanowires on the carbon cloth. (**e**,**f**) SEM images of hierarchical core-shell NiCo_2_O_4_@NiMoO_4_ nanowires on the carbon cloth.

The morphologies of CC@NiCo_2_O_4_, and CC@NiCo_2_O_4_@NiMoO_4_ are displayed in Fig. 2. Figure 2a shows the scanning electron microscope (SEM) image of bare carbon fibers before loading active materials. The diameter of the carbon fiber is around 10–15 μm, and the surface is smooth, which facilitates to deposit active materials on its surface. From Fig. 2b, we can see that the NiCo_2_O_4_ nanowires are uniformly distributed on the carbon fiber in the low-magnification microscopy. Figure 2c,d show that the aligned NiCo_2_O_4_ nanowires with average diameter of about 200 nm are vertically grown on the carbon fiber substrate. The NiCo_2_O_4_ nanowires backbone structure on the carbon cloth and the space between the nanowires provide space for the growth of NiMoO_4_ nanosheets. Figure 2e depicts that the surface of NiCo_2_O_4_ nanowires becomes rougher and larger after the secondary hydrothermal route. In the high-magnification image (Fig. 2f), we can see that the NiCo_2_O_4_ nanowires are decorated with many tiny NiMoO_4_ nanosheets. The diameter of the hierarchical core-shell NiCo_2_O_4_@NiMoO_4_ nanowires is about 600 nm, bigger than initial diameter of the NiCo_2_O_4_ nanowires. Besides, the NiMoO_4_ nanosheets can be also uniformly grown on the carbon cloth via the same hydrothermal route to form NiMoO_4_@CC integrated electrode (Fig. S2). The hierarchical core-shell NiCo_2_O_4_@NiMoO_4_ can obviously improve the mass loading of unit area on the carbon cloth substrate, increasing the specific areal capacitance of the binder-free integrated electrode. More importantly, the unique nanostructure decreases the resistance of electrons from the active materials to the conductive substrate, providing higher rate performance of the electrode. Finally, the core-shell hybrid structure has better cycling stability.

**Figure 3:**
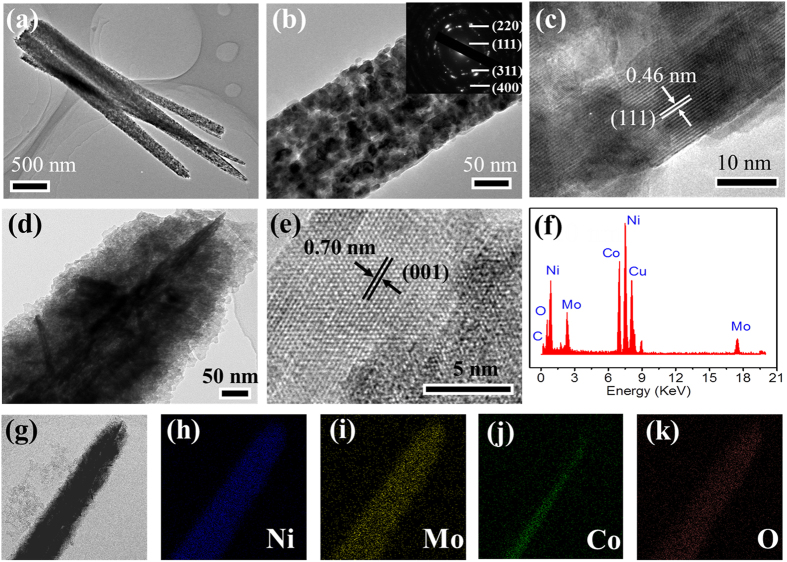
TEM and elemental analysis results of as prepared electrode materials. (**a**,**b**) TEM images of NiCo_2_O_4_ naowires. (**c**) HR-TEM image of a single NiCo_2_O_4_ naowire. (**d**) TEM image of an individual hierarchical core-shell NiCo_2_O_4_@NiMoO_4_ nanowire. (**e**) HR-TEM image of an individual NiMoO_4_ nanosheet outside. (**f**–**k**) EDS and elemental mapping of a single hierarchical core-shell NiCo_2_O_4_@NiMoO_4_ nanowire under TEM mode.

We used transmission electron microscope (TEM) and High-resolution TEM (HR-TEM) to further analyze the microstructure of the hierarchical core-shell NiCo_2_O_4_@NiMoO_4_ nanowires. Figure 3a–c present the TEM and HR-TEM images of NiCo_2_O_4_ nanowires backbone on the carbon cloth. From Fig. 3a, we can see that the nanowires of NiCo_2_O_4_ contain many nanoparticles and pores. Figure 3b indicates that the diameter of NiCo_2_O_4_ nanowires is about 100–150 nm. The selected area electron diffraction (SAED) in the inset of Fig. 3b clearly shows the polycrystalline structure of NiCo_2_O_4_ nanowires, further demonstrating that the nanowires are composed of nanoparticles. The well-defined rings are ascribed to (111), (220), (311) and (400) planes of cubic spinel NiCo_2_O_4_ (JCPDS no. 20-0781). From the HR-TEM image in Fig. 3c, we can see that interplanar spacing of the well-defined lattice fringes is 0.46 nm, corresponding to the (111) planes of NiCo_2_O_4_ nanowires on the carbon cloth.

Figure 3d reveals a typical TEM image of the NiCo_2_O_4_@NiMoO_4_ nanowire. The image shows obvious core-shell hybrid nanostructure. The tiny NiMoO_4_ nanosheets are uniformly grown along the NiCo_2_O_4_ wire, forming a hierarchical structure. In addition, the HR-TEM image in Fig. 3e indicates the interplanar spacing of 0.7 nm corresponding to the (001) planes of NiMoO_4_ nanosheets outside, confirming the core-shell structure. NiCo_2_O_4_ nanowires not only serve as active material in the integrated electrode, but also provide a fast electronic transfer path because of their higher conductivity. The backbone of NiCo_2_O_4_ wires is beneficial to the rate performance of the hierarchical core-shell NiCo_2_O_4_@NiMoO_4_ integrated electrode. We also analyzed the elements of the hybrid electrode material using EDS and mapping under the TEM mode. From Fig. 3f, we can see that the electrode material mainly contains Ni, Co, Mo, O elements, corresponding to the NiCo_2_O_4_ and NiMoO_4_ phase. Except for the four elements above, we can also find some Cu and C, which come from the Cu mesh substrate and carbon cloth fiber, respectively. The element mappings (Fig. 3g–k) clearly show that O and Ni are uniformly distributed within the whole hybrid electrode material, while Co and Mo are concentrated in the core and shell sites, respectively. So the element analysis further demonstrates that the hybrid electrode is a hierarchical core-shell nanostructure, in which NiCo_2_O_4_ nanowires and NiMoO_4_ nanosheets serve as core and shell, respectively.

X-ray photoelectron spectroscopy (XPS) was employed to further confirm the elements and the valence states in the integrated electrode, as shown in Fig. S3. For the Ni 2p core level spectrum (Fig. S3a), the peaks located at 872.9 and 879.7 eV correspond to Ni 2p_1/2_, while the peaks at 855.4 and 861.2 eV to Ni 2p_3/2_. The gap between Ni 2p_3/2_ and Ni 2p_1/2_ is 17.5 eV, indicative of the Ni^2^ oxidation state^51^. From Fig. S3b, the peaks at 233.4 and 236.5 eV are ascribed to the Mo 3d_5/2_ and Mo 3d_3/2_, respectively. The energy gap of 3.1 eV shows a Mo^6+^ oxidation state. For Co 2p, the peaks at 779.5 and 794.6 eV are accounted for the Co^3+^, while the peaks at 781.2 and 796.3 eV are due to the Co^2+^ oxidation state. The O 1s can be deconvoluted into O1, O2 and O3, which refer to the O-Co/Ni bonding, defect sites and physic-chemisorbed water, respectively^42^,^52^.

The porous characterization of NiCo_2_O_4_@NiMoO_4_ was measured by nitrogen adsorption and desorption at 77 K. Figure S4 shows that the Brunauer-Emmett-Teller (BET) surface area is 91.97 m^2^ g^−1^, which means that the electrode material has rich active surface to react with electrolyte ions. Furthermore, the corresponding pore size distribution calculated by Barrett-Joyner-Halenda (BJH) is about 30.8 nm, which can facilitate fast transfer of electrolyte ions in the electrode.

**Figure 4:**
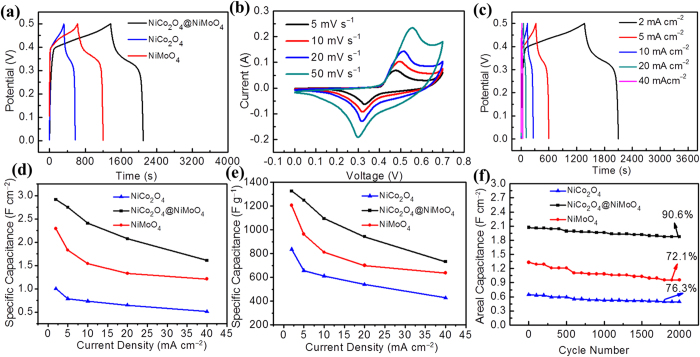
Electrochemical performance of as prepared CC@NiCo_2_O_4_@NiMoO_4_ integrated electrode. (**a**) Galvanostatic charge and discharge profiles of the CC@NiCo_2_O_4_@NiMoO_4_, CC@NiCo_2_O_4_, CC@ NiMoO_4_ integrated electrode at 2 mA cm^−2^. (**b**) CV curves of CC@NiCo_2_O_4_@NiMoO_4_ integrated electrode at various scan rates in the voltage window of 0–0.7 V. (**c**). Galvanostatic charge-discharge profiles of the CC@NiCo_2_O_4_@NiMoO_4_ integrated electrode at different current densities. (**d**,**e**) Areal and Mass specific capacitance of CC@NiCo_2_O_4_@NiMoO_4_, CC@NiCo_2_O_4_, CC@ NiMoO_4_ integrated electrode at different scan rates. (**f**) Cycling stability of CC@NiCo_2_O_4_@NiMoO_4_, CC@NiCo_2_O_4_, CC@ NiMoO_4_ integrated electrode at 20 mA cm^−2^.

The obtained CC@NiCo_2_O_4_@NiMoO_4_ sample was directly used as integrated electrode. The electrochemical performance was investigated in a three-electrode system. We chose 3 mol l^−1^ KOH as electrolyte, and the reference electrode and counter electrode were standard Hg/HgO and Pt foil, respectively. Figure 4a compares the charge and discharge curves of three integrated electrodes (CC@NiCo_2_O_4_, CC@NiMoO_4_, and CC@NiCo_2_O_4_@NiMoO_4_) at 2 mA cm^−2^. The result shows that the discharge time of CC@NiCo_2_O_4_@NiMoO_4_ is the longest, indicating its best specific capacitance. Cyclic voltammetry (CV) measurement was also carried out at various scan rates at the same potential window (see Fig. 4b). Apparently, the well-defined redox peaks in each CV profile are ascribed to the reversible faradaic redox reactions of Ni/Co-O, Ni/Co-O-OH with OH^−^. Besides, even at high scan rate of 50 mV s^−1^, the CV profile remains similar, implying excellent rate performance and chemical stability. Figure 4c presents the galvanostatic discharge curves at various current densities for CC@NiCo_2_O_4_@NiMoO_4_ electrode. We can see a discharge plateau at the voltage of 0.35 V, according to the CV redox peaks. The ASC of CC@NiCo_2_O_4_@NiMoO_4_ electrode is calculated based on eqn. (1) (see Methods). Remarkably, the ASC of as-prepared CC@NiCo_2_O_4_@NiMoO_4_ electrode is 2.917, 2.748, 2.406, 2.072 and 1.608 F cm^−2^ at current densities of 2, 5, 10, 20 and 40 mA cm^−2^, respectively. Moreover, the ASC can still keep to 55.1% of the initial value at 40 mA cm^−2^.

As comparisons, the ASCs of CC@NiCo_2_O_4_ and CC @NiMoO_4_ integrated electrode are 1.003 and 2.29 F cm^−2^ at 2 mA cm^−2^ (Fig. 4d), lower than those of CC@NiCo_2_O_4_@NiMoO_4_ integrated electrode. Figure 4e shows the mass specific capacitance (MSC) calculated by mass loading density according to eqn. (2) (see Methods). The MSC of the CC@NiCo_2_O_4_@NiMoO_4_ integrated electrode is 1325.9, 1249.1, 1093.6, 941.8 and 730.9 F g^−1^, respectively. The values are also higher than CC@NiCo_2_O_4_ and CC@NiMoO_4_ electrodes at the same testing condition. The electrochemical performance of the CC@NiCo_2_O_4_@NiMoO_4_ electrode is better than most of the reported individual binary metal oxides like NiCo_2_O_4_ nanowires (1283 F g^−1^ at 1 A g^−1^)^36^, NiMoO_4_ nanowires (1.27 F cm^−2^ at 5 mA cm^−2^)^41^, and NiMoO_4_ nanosheets (1221.2 F g^−1^ at 1 A g^−1^)^43^.

Figure 4f presents the cycling performance at 20 mA cm^−2^. The results show that the ASC of CC@NiCo_2_O_4_@NiMoO_4_ integrated electrode can remain 90.6% of the initial value even after 2000 cycles, much higher than those of the CC@NiCo_2_O_4_ (76.3%) and CC@NiMoO_4_ integrated electrode (72.1%). This high cycling performance can be explained by the synergic effect of NiCo_2_O_4_ and NiMoO_4_. Besides, the core-shell hierarchical nanostructure has better structure stability during the cycling process.

**Figure 5:**
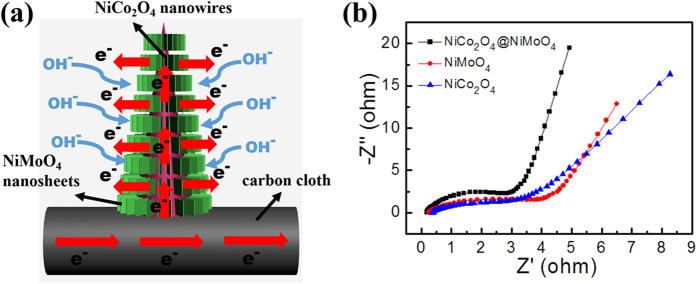
(**a**) Schematic transport and redox reaction process of electrons and ions in CC@NiCo_2_O_4_@NiMoO_4_ integrated electrode. (**b**) Electrochemical impedance spectra of CC@NiCo_2_O_4_@NiMoO_4_, CC@NiCo_2_O_4_ and CC@ NiMoO_4_ integrated electrodes.

Such high ASC and rate performance of the CC@NiCo_2_O_4_@NiMoO_4_ integrated electrode demonstrate the great advantage of the hierarchical core-shell nanostructure. The schematic transport and redox reaction process for electrons and electrolyte ions in the electrode is illustrated in Fig. 5a. Because NiCo_2_O_4_ has higher electronic conductivity, so electrons can efficiently transfer from the carbon cloth to the NiMoO_4_ nanosheets via the backbone of the NiCo_2_O_4_ nanowires. So this integrated electrode has sufficient electrons to participate in the redox reaction. Furthermore, the ions can penetrate into the NiMoO_4_ nanosheets to react with the inner NiCo_2_O_4_ nanowires to improve the electrochemical performance of the whole integrated electrode. Finally, the hierarchical core-shell nanostructure has better stability, so it cannot be easily destroyed during the redox reaction process.

Electrochemical impedance spectroscopy (EIS) was used to analyze the electrochemical mechanism. Figure 5b shows the Nyquist plots of CC@NiCo_2_O_4_@NiMoO_4_ in the frequency range of 0.01–100 kHz. As can be seen, the CC@NiCo_2_O_4_@NiMoO_4_ electrode exhibits the lowest charge transfer resistance (*R*_ct_), suggesting the largest efficient electro-active surface area. Furthermore, the diffusion resistance of the electrode is also lowest, indicating the fastest electrolyte ion diffusion during the redox reaction.

In conclusion, a two-step hydrothermal route has been carried out to fabricate hierarchical core-shell NiCo_2_O_4_@NiMoO_4_ nanowires grown on carbon cloth as electrode for faradaic supercapacitors. The fabricated CC@NiCo_2_O_4_@NiMoO_4_ integrated electrode shows an enhanced capacitance of 2.917 F cm^−2^ at 2 mA cm^−2^ and excellent cyclability with 90.6% retention over 2000 cycles. The superior faradaic capacitance can be accounted for the rational core-shell hierarchical nanostructure and the synergic of NiCo_2_O_4_ nanowires and NiMoO_4_ nanosheets. We believe that the design and synthesis method can open up a promising and efficient way to develop new electrode materials for high-performance supercapaitors.

## Methods

### Materials synthesis

CC@NiCo_2_O_4_@NiMoO_4_ integrated electrode was fabricated by a two-step hydrothermal route followed by thermal annealing in inert gas. Firstly, the uniform NiCo_2_O_4_ nanowires were grown on the carbon cloth through a facile hydrothermal reaction to form CC@NiCo_2_O_4_ nanowires. Then NiMoO_4_ nanosheets were synthesized based on the backbone of NiCo_2_O_4_ nanowires to fabricate the CC@NiCo_2_O_4_@NiMoO_4_ integrated electrode by a secondary hydrothermal reaction. In a typical process, 2 mmol Co(NO_3_)_2_·6H_2_O, 1 mmol Ni(NO_3_)_2_·6H_2_O and 9 mmol urea were dissolved into 50 ml distilled water to achieve a clear pink solution. A piece of carbon cloth was pretreated by ultrasonic in deionized water and absolute ethanol each for 30 min. Then the cleaned carbon cloth was placed in the solution. The hydrothermal temperature and time were to 120 °C and 6 h, respectively. The coated carbon cloth was cleaned by deionized water and ethanol, and then dried in oven at 80 °C for 12 h. Finally, the sample was annealed in pure N_2_ atmosphere at 400 °C for 3 h to obtain CC@NiCo_2_O_4_ integrated electrode.

A secondary hydrothermal reaction was adopted to grow the NiMoO_4_ nanosheets on the backbone of NiCo_2_O_4_ nanowires to fabricate the CC@NiCo_2_O_4_@NiMoO_4_ integrated electrode. Briefly, 1 mmol Na_2_MoO_4_·2H_2_O and 1 mmol Ni(NO_3_)_2_·6H_2_O were dissolved in 50 ml mixed solvent (the volume ratio of distilled water and absolute ethanol is 1:1) to achieve a precursor solution, and then put into a stainless steel autoclave. The as-preparedCC@NiCo_2_O_4_ integrated electrode was sealed in the autoclave, and kept at 130 °C for 6 h. The resulting sample was taken out and rinsed with deionized water and ethanol for several times, followed by annealing at 400 °C for 2 h in pure N_2_ to obtain CC@NiCo_2_O_4_@NiMoO_4_ integrated electrode. We also fabricated CC@NiMoO_4_ integrated electrode with the same condition.

### Material Characterizations

XRD (Panalytical X’pert PRO, Holland, Cu K_αl_ irradiation, *λ* = 1.5406 Å, scan rate: 5° min^−1^) was performed using a multi-purpose diffractometer to analyze the phase. The field-emission SEM (FE-SEM, FEI Sirion200, Holland) was used to characterize the morphology of the products. The microstructure and element analysis were characterized using TEM (JEOL JEM-2010F, Japan) coupled with an EDS X-ray spectrometer (Oxford Instrument). XPS measurement was carried out using Al K_α_ X-ray. The BET surface area, pore size and volume were measured by N_2_ absorption on a Micromeritics ASAP 2020 analyzer.

### Electrochemical measurements

Electrochemical measurements were carried out in a three-electrode configuration on CHI760E electrochemical workstation (Chenhua, Shanghai). The binder-free CC@NiCo_2_O_4_@NiMoO_4_ was directly used as working electrode, and the nominal area was about 1 cm^2^. A Hg/HgO electrode and high-pure Pt foil served as reference and counter electrode, respectively. The electrolyte was 3 mol l^−1^ KOH. The mass-loading of CC@NiCo_2_O_4_, CC@NiMoO_4_, and CC@NiCo_2_O_4_@NiMoO_4_ were 1.2, 1 and 2.1 mg cm^−2^, respectively.

Electrochemical impedance spectroscopy (EIS) was measured using an alternating-current (AC) voltage with 5 mV, and the frequency was 0.01–100 kHz. The specific capacitance was calculated by equation (1) and (2).


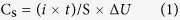



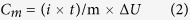


where C_s_ and *C*_*m*_ are the areal and mass capacitance of the active material supported on the carbon cloth, respectively. *i* is the discharge current applied to the electrode (A); *t* is the discharge time from high to low potential (s) and Δ*U* is the gap of high and low potential (V).

## Additional Information

**How to cite this article**: Huang, L. *et al*. Hierarchical core-shell NiCo_2_O_4_@NiMoO_4_ nanowires grown on carbon cloth as integrated electrode for high-performance supercapacitors. *Sci. Rep.* **6**, 31465; doi: 10.1038/srep31465 (2016).

## Supplementary Material

Supplementary Information
