# Supplementary material for: Hierarchical core-shell NiCo2O4@NiMoO4 nanowires grown on carbon cloth as integrated electrode for high-performance supercapacitors
Source: Sci Rep. 2016 Aug 12;6:31465. doi: 10.1038/srep31465 (PMC4981856; doi:10.1038/srep31465)
Supplement: Supplementary Information [file srep31465-s1.doc]

**SUPPLEMENTARY INFORMATION FOR**

Hierarchical core-shell NiCo_2_O_4_@NiMoO_4_ nanowires grown on carbon cloth as integrated electrode for high-performance supercapacitors

Liang Huang, Wei Zhang, Jinwei Xiang, Henghui Xu, Guolong Li and Yunhui Huang*

State Key Laboratory of Material Processing and Die & Mould Technology, School of Materials Science and Engineering, Huazhong University of Science and Technology, Wuhan 430074, P. R. China.

Correspondence and requests for materials should be addressed to Y.H.H. ([huangyh@hust.edu.cn](mailto:huangyh@hust.edu.cn))

**Supplementary**

**Supplementary Figures:**


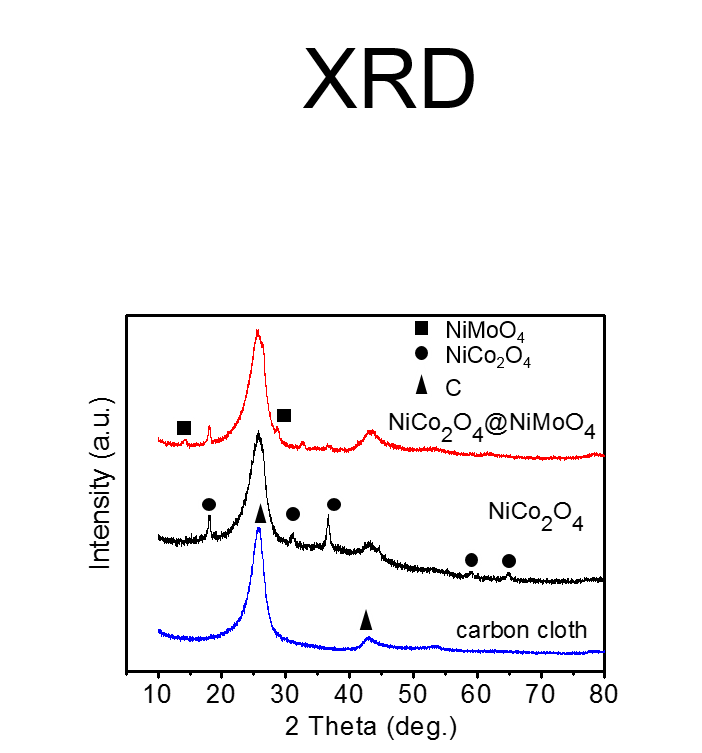


**Figure S1. Typical XRD pattern of carbon cloth, CC@NiCo_2_O_4_, and CC@NiCo_2_O_4_@NiMoO_4_.**


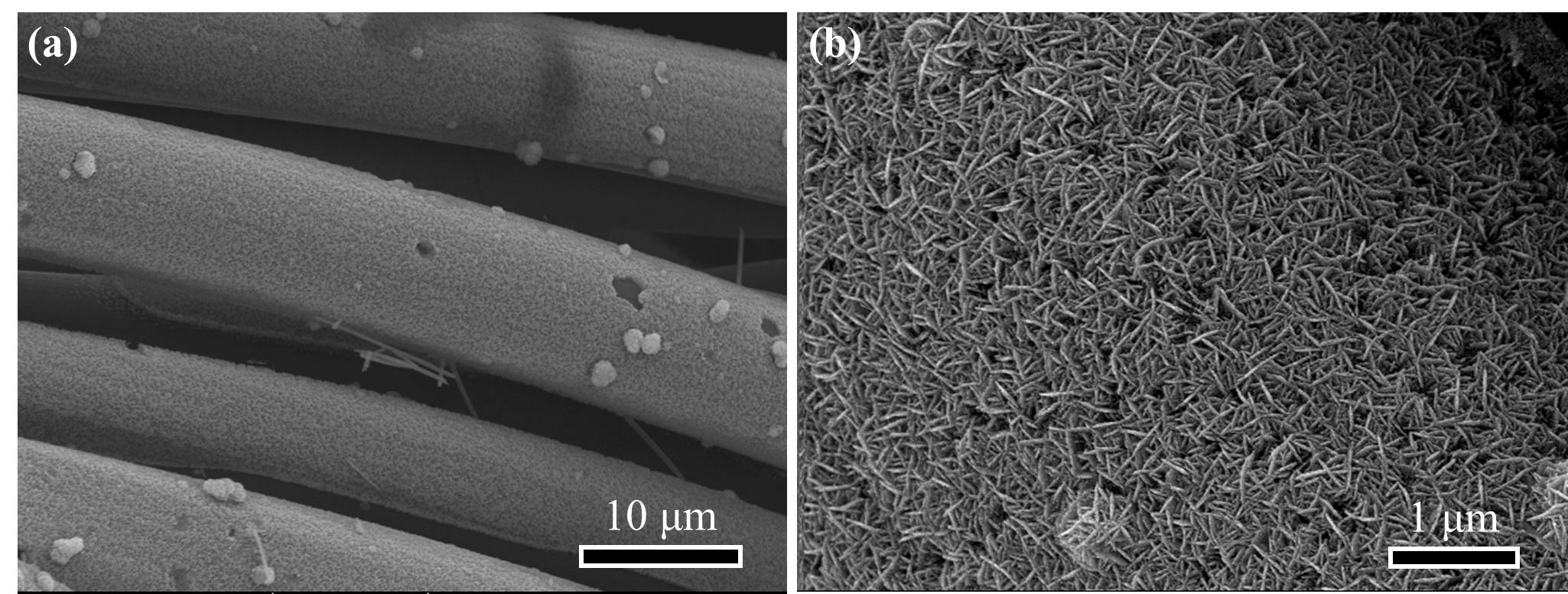


**Figure S2. SEM images of NiMoO_4_ nanosheets supported on the carbon cloth.**


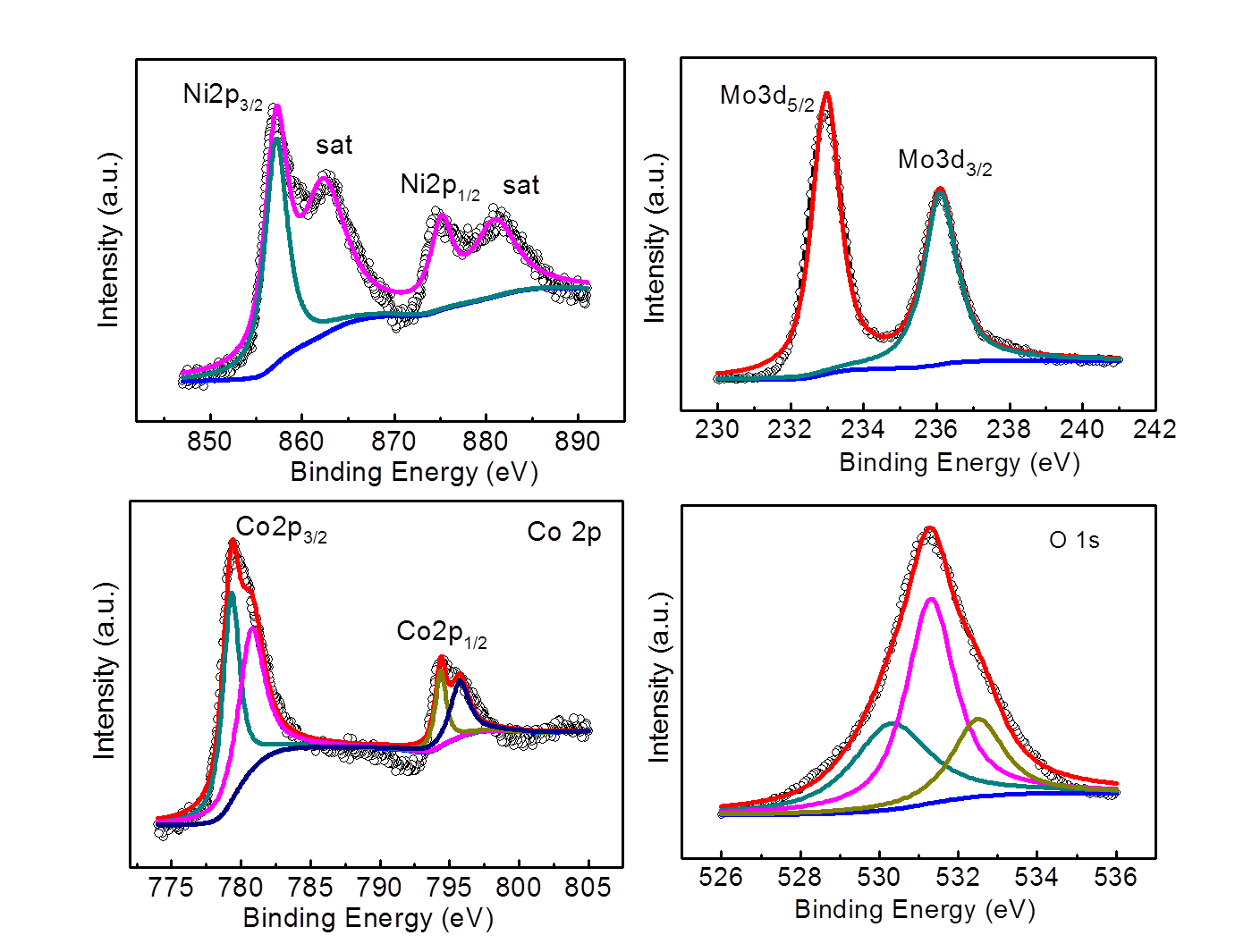


**Figure S3. XPS analysis of hierarchical core-shell NiCo_2_O_4_@NiMoO_4_ nanowires.**


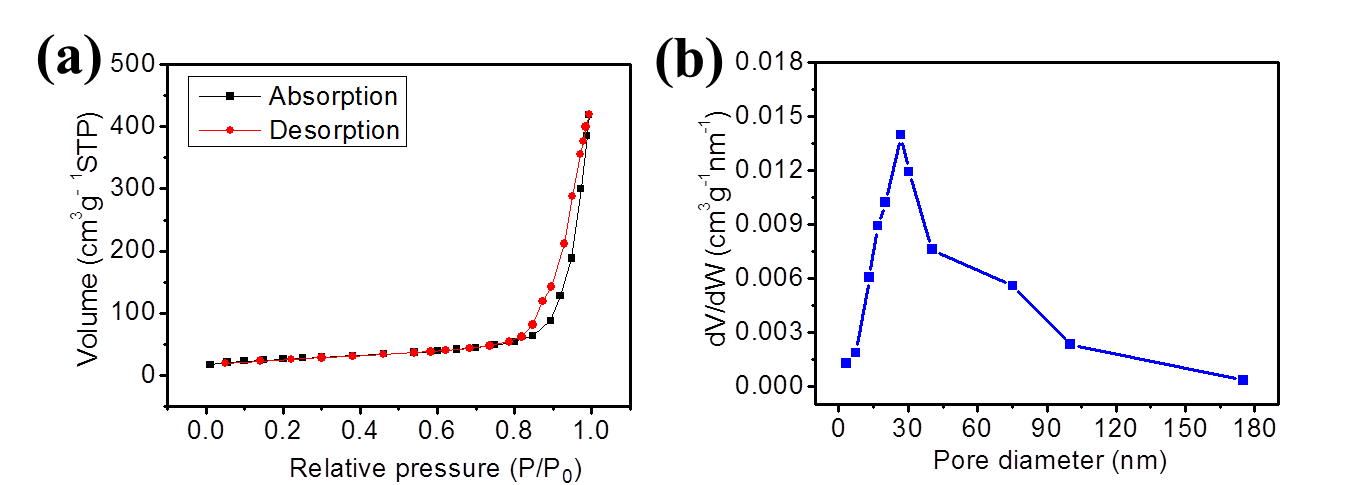


**Figure S4.** (a) N_2_ adsorption-desorption isotherm of the CC@NiCo_2_O_4_@NiMoO_4_ integrated electrode. (b) The pore size distribution of the CC@NiCo_2_O_4_@NiMoO_4_ integrated electrode obtained from adsorption branches of corresponding isotherms by BJH method.
